# Demographic profile of physician participants in short-term medical missions

**DOI:** 10.1186/s12913-016-1929-x

**Published:** 2016-12-07

**Authors:** Paul H. Caldron, Ann Impens, Milena Pavlova, Wim Groot

**Affiliations:** 1 Maastricht Graduate School of Governance, University of Maastricht, Maastricht, Netherlands; 2 Midwestern University Institute of Healthcare Innovation, 555 31st Street, 60515 Downers Grove, IL USA; 3 Department of Health Services Research; CAPHRI, Maastricht University Medical Center, Faculty of Health, Medicine and Life Sciences, PO Box 616, 6200 MD Maastricht, Netherlands; 4 Department of Health Services Research; CAPHRI, Maastricht University Medical Center, Faculty of Health, Medicine and Life Sciences, Room 0.033, Duboisdomein 30, 6229 GT Maastricht, Netherlands

**Keywords:** Medical missions, Short-term, Volunteerism, Demographics

## Abstract

**Background:**

The US is the leading sending country for short term medical missions (STMMs), an unregulated and unsanctioned, grass roots form of direct medical service aid from richer countries to low and middle income countries. The objective of this study is to profile US physicians who go on such missions by means of a survey sample of the US physician population.

**Methods:**

An online survey solicited information on physician participation in STMMS as well as demographic and professional features. Responses were descriptively tabulated and multivariate regressions were performed to model for physician profiles related to STMM participation.

**Results:**

Physician participants in STMMs are more likely to be a surgeon, anesthesiologist or pediatrician, married with few or no children at home, later in their career and have an income of $200–250 K.

**Conclusions:**

Specialty is the strongest predictor of participation. STMM participation does not differ by race, ethnicity nor religion. Descriptive statistics further provide a limited profile of participants. Direct expenses may have less influence on participation than opportunity costs. Potential clues about motivation that may be inferred from the features of the profile are discussed.

**Electronic supplementary material:**

The online version of this article (doi:10.1186/s12913-016-1929-x) contains supplementary material, which is available to authorized users.

## Background

The activity wherein physicians and other medical workers from higher income countries go abroad for pre-planned periods of days to weeks in order to provide uncompensated direct care to persons in lower and middle income countries (LMIC)^1^ weeks has been commonly referred to, among other terms, as short-term medical missions (STMMs). These excursions are distinguished from full-time relief employment and ad hoc responses to domestic or external disasters.

Through literary extractions reported in their systematic review in 2012, Martiniuk et al. identified the USA as the leading sending country for STMMs followed by Canada, United Kingdom and Australia in terms of numbers of missions [1]. Other reviews have comparatively analyzed surgical mission platforms (permanent facilities vs. hospital ships vs. mobile units), data aggregation from STMM related literature, and social, economic and diplomatic aspects [2–4]. We have found no reports that specifically profile physicians who perform STMMs in terms of demographic or professional characteristics. A mail survey of surgeons affiliated with one North Carolina hospital system that did not distinguish between domestic and international volunteering, found full time practice as the only demographic correlate with volunteering; not age, gender, or surgical subspecialty [5].

The aim of this study is to profile the STMM physician participant using response data from the Physicians’ Giving Back Survey (PGBS) with respect to natural, professional, family and economic dimensions. Our exploration sought the demographic profile of “who” goes on uncompensated medical service trips in the prospect that it may hold some insights into “why”, beyond the assumed general mechanisms of altruism that drive conventional forms of philanthropy and volunteerism [6–9].

Without theoretical underpinnings or precedent literature as a guide, we constructed a set of explanatory variables that were as comprehensive as feasible, avoiding the practical challenge of a survey too lengthy or complex for busy physicians to want to click through. The final set includes a blend of typical census demographics and professional characteristics. We investigated if the stable, unchosen traits from nature and nurture such as gender, race, ethnicity, religion or being born in the US generally have more bearing on participation than more changeable life features like marital status, children at home, city population size, years in practice, practice situation and age group. Could region be an influence on STMM participation, related to the traditional image of the various parts of America: the Southern “Bible Belt”, the Midwestern “work ethic”, and coastal diversity or liberalism? Predicting who will repeat an activity may be intrinsic to the understanding of motivation. Our objective is to identify the characteristics that might impel a physician to go, not to define factors of poorer country need or social ties that may draw US physicians there, other than what may be inferred from where in the world they go.

The marketing theoretical and research literature that links the “who” in demographics to “what” individuals do, buy, or watch is vast. The “what” in our study is the act of participation in STMMs. Though such a link to “why” is more tenuous, we will speculate on what the profile infers about motivations.

## Methods

Survey methodology for the PGBS has been previously described [10] (Additional file 1). The PBGS was designed to gather information from US-domiciled physicians who had completed all formal general and specialty training on their participation in a finite list of philanthropic and volunteer activities along with the physicians’ demographic characteristics. The PGBS was conducted as an online survey in 2014 in the US. Beta testing of the PGBS was performed utilizing a selected group of 15 identified physicians whose critiques were incorporated into the final PGBS version. Exempt status was granted for use of human subjects for the survey from the Investigational Review Board of Midwestern University Office of Research and Sponsored Programs, Downers Grove, Illinois, USA. The proprietary email database of Healthcare Data Solutions (HDS) was used for the survey contacts. The HDS database conforms to industry best practice guidelines for business-to-business email acquisition, adheres to US CAN-SPAM guidelines and maintains a quarterly “permissioning” and validation process. The survey was implemented through SurveyMonkey©. Deployment of the survey to 109,237 unique physician emails was executed between January 30 and February 27, 2014. Response reception was closed on 30 April 2014. The email list included only physicians who were licensed to conduct the full spectrum of medicine (US MD, IMG, and DO).^2^ The survey targeted 93% MDs/IMGs and 7% DOs, proportionate to the US physician population distribution.^3^ The survey was disseminated equally to the four regions of the US^4^ in close proportion to the specialties practiced by the US physician population [11]. HDS’s DirectSelect tool herein eliminated titles such as Doctor of Chiropractic, Doctor of Optometry, Doctor of Podiatric Medicine, Licensed Acupuncturist, Naturopathic Doctor, dentists and PhDs.

The first question of the survey screened for target sample of physicians that had completed all formal training and are or had been in practice in the US. Respondents who subsequently affirmed STMM participation were directed to alternate pathways dependent upon single-mission versus multiple-mission participation. Income data was collected along with respondents’ demographic and professional characteristics. IBM SPSS version 22.0 was utilized in the one-sample chi2 test comparisons of sample and population characteristics. STATA version 12 and Excel® proprietary software were utilized throughout the analysis of data.

Age of responders was re-coded for groupings of 25–39, 40–55 and 56–73 years to assess for an age, and thereby a life and career stage, effect. Marital status was simplified to those who were legally married or not. For the analysis, specialties were aggregated into four broad categories including adult medicine, pediatric medicine, surgery and anesthesia, and other respondent specialties (psychiatry, pathology, radiology, nuclear medicine, dermatology, pain medicine). States of domicile were aggregated into the four regions for the regressions. Models were considered significant if a Prob > chi2 of at least 0.10 was demonstrated and the coefficients of the explanatory variables were considered significant if the *P* < = 0.05 (95% CI).

For the purpose of comparing the incomes of our STMM participant sample and US physician population incomes, we aggregated data on incomes of the 35 most common general practice and specialty categories from the Medical Group Management Association (MGMA) 2013 compensation report and matched these with specialty population numbers from the Physician Specialty Data Book 2013 data [12, 13].

Cross-tabulations in STATA were executed to compare the crossover rate of surgeons and anesthesiologists going on purely medical STMMs and adult and pediatric medical physicians going on surgically-focused STMMs.

## Results

### Descriptive characteristics of respondents

In total, 631 surveys were completed, a “click through” rate of 0.62% consistent with typical response rates to email surveys generated to physicians from the proprietary HDS database (January 2014: 25th percentile 0.17, 75th percentile 0.72) (personal communication with HDS). Data from 601 (response rate 0.55%) fit the target criteria of being physicians who had completed all training were therefore included in the analysis. The one-sample chi square test used for the comparison of the PGBS respondent sample to the US physician population showed statistical comparability with respect to the three demographic characteristics of race, civil status, type of medical degree, and non-comparability with respect to the five demographic characteristics of gender, age, IMG status, religion, and region of the country [10]. The top 16 of 29 respondent specialties displayed a similar rank order to the top 16 of the standard US physician specialty classifications [10, 11].

Thirty-two percent of respondents indicated that they had participated in at least one STMM after completion of all training. Seventy-seven percent of mission participants had gone on more than one STMM. STMM participation prevalence (32%) exceeded pro-bono participation in local free or sliding scale clinics (17.5%), domestic short term missions (9.3%) and domestic disaster relief (8.8%), but less with those who indicated that a system was in place in their practices to accommodate patients with limited means to pay (39.3%). STMM length was provided for 908 of the total 926 STMMs reported and averaged 11.8 days in total (range 1–90 days, SD 10.3 days). Effectively, the mean duration STMM would require the physician to be absent from gainful practice for 2 weeks; if on average, physicians are remuneratively active 46 weeks yearly, then the mean loss may be 4.3% of annual income during the year they went on one mission [14].

Sixty-five percent of physicians responding to the PGBS were in the upper age category (56–73 years) (Table 1). More physicians in this category (35%) were mission participants than the younger two age groups; indeed, this oldest age grouping comprised 72% of all responders who had gone on an STMM. Other demographic characteristics of survey responders and the subset of STMM participants are further tabulated in Table 1.

**Table 1:** Demographic characteristics of mission participants and survey respondents

Demographic	Variable Category	Survey Respondents (*N* = 601) Percent (%)	Mission Participants (*N* = 192) Percent (%)	
Age	25–39 years	4	3	
	40–55 years	31	25	
	56–73 years	65	72	
Gender	Male	58	64	
	Female	42	36	
Race	White	82	79	
	Asian	11	12	
	Black/African American	3	6	
	Native Hawaiian/Pacific Islander/Other	4	5	
Hispanic Ethnicity	Hispanic	5	5	
Naturalization	Born in USA	78	76	
	Born outside USA	22	24	
Medical Training	Med School - USA	81	80	
	Med School - abroad	19	20	
	Post-grad - USA	97	97	
	Post grad - abroad	3	3	
Marital status	Unmarried	20	25	
	Married	80	75	
Religion	Christianity	49	53	
	None	21	20	
	Judaism	19	16	
	Buddhism/Hinduism/Islam/Other	11	11	
Region of US	Northeast	33	28	
	Southern	18	21	
	Midwest	22	24	
	West	26	24	
Children	Children, biologic and/or adopted	88	90	
	Mean number of children	2	2	
	SD	2	2	
	Range	0–13	0–6	
Years in practice	Mean	24	17^a^	11^b^
	SD	11	12^a^	10^b^
	Range	0–50	0-43^a^	0-36^b^

^a^At only STMM

^b^At first of multiple STMMs

**Fig. 1:**
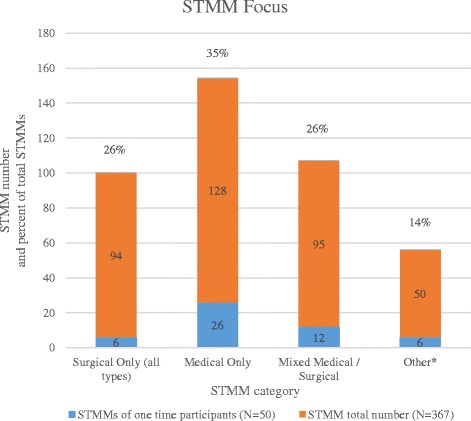
Mission focus by category

Respondents who indicated STMM experience were segregated through the PGBS algorithm as having gone on a single mission versus multiple missions. In both subsets, where category of mission was specified (*N* = 367 of 928), the most frequent STMM focus was medical (non-surgical) (Fig. 1). The proportion of surgical STMMs to medical STMMs is 26 and 35% respectively, leaving aside those categorized as mixed medical / surgical STMMs. Cross-tabulations in our sample of STMM participants reveal a 15.2% cross-over by surgeons/anesthesiologists to medical missions (33% in single STMM participants and 10% in multiple STMM participants) and a 5% cross-over by adult and pediatric medicine physicians to surgical missions.

**Fig. 2:**
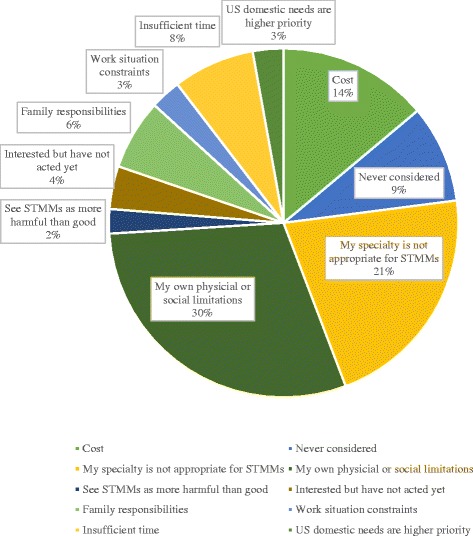
Reasons for primary non-participation in STMMs (N-354)

Respondents who had not participated in STMMs most commonly cited physical or social limitations (any medical or regulatory barriers to traveling abroad in the view of the physician) and practicing a specialty that is not a good fit for LMIC missions more frequently than cost constraints as the reason for non-participation (Fig. 2). The remainder of the leading five reasons cited for primary non-participation included time, family, and work situation constraints respectively. Twelve percent felt that US domestic needs took priority over those abroad. Principal reasons given by single mission participants who chose not to repeat (secondary non-participation; 10.9%) were expense and time away from spouse or family.

### The stmm physician profile – multivariate testing

Using any mission participation (0 = no, 1 = yes) and single versus multiple mission participation (0 = single, 1 = multiple) as binary dependent variables, multivariate logit regressions controlled for seventeen explanatory variables that can be conceptually aggregated in two domains. The first domain includes typically stable demographic characteristics including gender, race, Hispanic ethnicity, religious affiliation, degree (MD/DO), being born in the US, having completed medical school in the US, specialty (having completed most post graduate training in the US) and income level. The second domain includes those characteristics that commonly change over time including region of the US, marital status, children in the home, city population size, cumulative years in practice, practice situation (solo practice, academic, hospital-based, government or public facility, multi-specialty group, single-specialty group) and age group. Lastly, other general types of charitable and volunteer activities typically performed by physicians (giving money or donations-in-kind, pro-bono medical teaching, pro-bono domestic direct medical care, donating time to professional or patient support organizations and having a system in one’s practice to accommodate those with limited ability to pay) were assessed as binary explanatory variables in separate regressions. Respondents who provided no demographic data (*n* = 100, 16.6%) were omitted from the regression models. Five hundred one respondents who completed nearly all questions were included in the regressions. For seven independent variables where more than 4 observations were missing, including region (*n* = 9), children (*n* = 9), practice situation (*n* = 9), marital status (*n* = 13), religion (*n* = 24), city population (*n* = 27), and highest taxable income (*n* = 54), non-reporting variables were used. Development of indicator variables to account for all missing observations for all 601 respondents did not alter the effects of explanatory variables on response variables except that participation in other charitable activities would predict STMM participation. Reduction of the model, carried out through serially withdrawing explanatory variables whose significance exceeded a P-value less than 0.10, also did not alter the results of the regressions using original variables. The significant predictors found in these three regression models are combined in Table 2.

**Table 2:** Significant results of multivariate logit regressions for any STMM participation, repeated STMM participation, and correlation with other volunteering or philanthropic activities

Any STMM participation (0 = no, 1 = yes) with demographic and professional characteristics (Observations 501, Prob > chi2 = 0.0000)	Coef.	*P* > z	[95% Conf. Interval]		Odds Ratio	[95% Conf. Interval]	
Ages 25–39	−1.33401	0.046	−2.64383	−0.02419	0.2634195	0.0710885	0.9761046
Ages 40–55	−0.7503	0.019	−1.37846	−0.12214	0.4722246	0.2519665	0.8850227
Pediatric cognitive medicine	1.128995	0.002	0.417094	1.840897	3.092548	1.517546	6.302186
Surgery/anesthesiology	1.059275	0.000	0.54091	1.577639	2.884278	1.717569	4.843509
Married	0.661369	0.02	0.104369	1.218368	1.937442	1.11001	3.381663
Highest taxable income not reported	−1.45118	0.004	−2.45179	−0.45057	0.2342945	0.08614	0.637267
Single STMM (=0) Multiple STMM (=1) with demographic and professional characteristics (Observations 193, Prob > chi2 = 0.0891)	Coef.	*P* > z	[95% Conf. Interval]		Odds Ratio	[95% Conf. Interval]	
Surgery/anesthesiology	1.111934	0.029	0.111945	2.111924	3.040233	1.118451	8.264127
Any STMM participation (0 = no, 1 = yes) with other types of volunteering and philanthropy (Observations 501, Prob > chi2 = 0.0145)	Coef.	P > z	[95% Conf. Interval]		Odds Ratio	[95% Conf. Interval]	
System in practice to accommodate those who have limited ability to pay	−0.60334	0.003	−1.00231	−0.20436	0.5469826	0.3670295	0.8151659

On the basis of coefficients as well as odds ratios, respondents in the age groups 25–39 and 40–55 were less likely to report STMM participation than those in the age group 56+. Further, in our sample, the US physician who volunteers for STMMs is more likely to be a pediatrician, surgeon or anesthesiologist, married and more likely than non-participants to report their highest taxable income in our survey. Participants and non-participants were not distinguishable on the basis of other characteristics in either the static or changing domain. In the separate regression using other charitable activity options as binary independent variables, STMM participants are unlikely to have a system in their practices to accommodate those with limited ability to pay. No other philanthropic or volunteering activities appeared to have an influence on STMM participation.

The regression to distinguish single from multiple STMM participants controlling for the same set of characteristics indicated only specialty, i.e., surgical or anesthetic practice, as having an influence on the likelihood of repeated STMM participation. A logit regression controlling for other philanthropic and volunteer activities and repeated STMM participation did not produce a significant model.

### Additional implications of pgbs responses on attributes of stmm participants

**Fig. 3:**
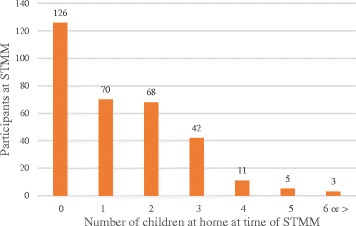
Number of children at home at time of STMM

Having children at the time of the survey (88%) did not predict STMM participation in multivariate regressions. Nonetheless, descriptive observation suggested that the mode number of children at home at the time of an STMM was zero for 38.8% of participants regardless of mission number. Sixty percent of STMM participants had no more than one child at home at the time of a mission and participation decreased consistently with increasing numbers of children at home (Fig. 3).

**Fig. 4:**
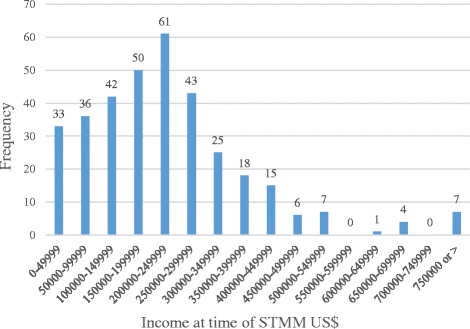
Frequency distribution - Income at time of STMM

The regressions on STMM participation and repetition indicate that highest taxable income level at the time of the survey was not predictive of such participation. Figure 4 illustrates however that the most common highest taxable income range of STMM participants at the time of STMM was $200,000–249,999 (mean estimated from the weighted midpoints of ranges $256,077, SD $324,038). Although there are no bright line income thresholds at which physicians begin to take part in STMMs or cease to participate, there appears to be increasing participation as incomes approach the mean, then little participation beyond incomes of about $500,000. The statistics in Fig. 4 are similar to a composite of primary care and specialty income levels from Medscape physician survey respondent samples for the US physician population in 2014 that vary somewhat by region [15, 16]. Both the two year running average of groupings of 100,000 USD and totals from 3 larger groupings in our aggregated data from the MGMA 2013 compensation report and the Physician Specialty Data Book 2013 (see Methods section) would place the overall US physician population income mean in the US$300,000–450,000 range with a less positive skew compared to the curve of STMM participants seen in our sample [12, 13].

Of 926 STMMs reported by respondents, 60% were mission trips to countries in Latin America (*n* = 552) followed by Africa (*n* = 129) as the most commonly visited. The remaining STMMs were regionally distributed as follows: Africa 14%, Southeast Asia 11%, the Indian subcontinent 9%, 2% each for Eastern Europe and the Pacific Islands, and 1% each for the Middle East and Central Asia. Seventy-six percent of STMM participants in the sample were non-Hispanic whites. These physicians carried out 60% of their STMMs in Latin American countries. Ninety-five percent of STMMs by participants who identified themselves as black or African American were carried out in Latin America and only 5% in Africa. In contrast, Hispanic physicians performed 85% of their STMMS in Latin America. Fifty-seven percent of STMMs by Asian physicians occurred in Asian countries (Central Asia, Southeast Asia and the Indian subcontinent) and another 25% in Latin America. Non-white Hispanics, for example Puerto Rican US citizens of African descent, were not seen in the sample.

## Discussion

Initial descriptive statistics from the respondent sample do not project a stark profile of the physician who participates in STMMs. Nonetheless, from the regressions and trends in descriptive statistics, an image begins to emerge of a more mature surgeon, anesthesiologist or pediatrician, married with few or no children at home, with a lower than average annual income of around $250,000. The features of this image may render clues to why physicians go. Between the domains of stable and changing characteristics, the life choices physicians make seem to tell us more than their innate traits or origins do about who is likely to participate.

More than any other single characteristic, STMM participation is influenced by specialty. Overall, surgeons and anesthesiologists are the most likely specialty categories to participate. This finding is consonant with our recent systematic literature review wherein we established that most articles providing data, guidelines and instructions regarding STMMs are found in specialty surgical literature, presumably because surgical missions tend to provide a finite set of discrete procedures more amenable to process and outcome assessments [4]. Global health measures such as water sanitation, mosquito net implementation, and HIV prevention can reduce a broad swath of illness, but for surgical disorders such as cataracts, cleft palates and fistulas, only one-to-one interaction with a skilled professional will help, making both the need for and the benefits accruing from surgical volunteering in underserved areas stand apart. Counterintuitively, a greater portion of missions have a medical rather than surgical focus suggesting that medical physicians are at least as much drawn to pro-bono missions, even if the effects on local disease burden are less precisely measurable than for surgical procedures. At the same time, cross-tabulations show a 15.2% cross-over to medical missions by surgeons/anesthesiologists and a 5% cross-over to surgical missions by medical physicians. It might be that some surgeons and anesthesiologists go on medical mission trips at times perhaps to connect back to primary care skills. US physicians in our sample who participated in any STMM are more likely to be in the latter rather than earlier years of professional life, in procedurally oriented specialties or pediatrics. Participation later in career may not be simply a function of longer experience, but perhaps that physicians in earlier years of practice must concentrate on a successful career start-up, and those in middle years bear more administrative responsibilities at work and have more children at home. Participation could depend less on youthful idealism and vitality, and more on these later career and life stage elements. Pediatricians in the US remain in the lower tiers of professional income [12]. The choice to participate in unpaid STMMs by these doctors would appear to be consistent with non-monetary gratification, that is, less related to income and less sensitive to forgoing income by spending time on missions. Children in poverty may perhaps evoke relatively more sympathy and appear to have more need than adult targets of pro-bono care, thus triggering a response to a receiver-side characteristic. Surgeons and anesthesiologists, as are other procedurally-oriented physicians in the US, exist on the higher end of the physician income spectrum [12]. Such procedural orientation further lends itself to missions that encompass a few discrete procedures that can be selective and scheduled a priori.

Another counterintuitive suggestion from our regressions is that STMM participants are less likely than non-participants to have a system in place in their practices to accommodate those with limited ability to pay. Wherein physicians are willing to give their time and talent in charitable circumstances for no compensation at all (and even at substantial personal cost), should we interpret that in their own practices they would maintain a stricter policy for payment and thereby clearly demarcate where they do business and where they do charity?

In the regression models, income was not seen to predict participation in STMMs. No shelf is seen in the distribution curve to indicate a threshold income allowing for or dissuading STMM participation. Nonetheless, one interpretation of the positive skew of the curve of Fig. 3 is that opportunity cost incurred at higher incomes is more influential than out-of-pocket costs, since opportunity cost sensitivity would be positively correlated with income generating potential. This may suggest a sort of “price elasticity” for the non-monetary rewards of pro-bono work. Decreasing participation with increasing income in the sample cannot be solely attributed to effects of aging or seniority since the sample showed relatively higher participation by older physicians. US physician incomes appear to plateau relative to the market early, i.e., as suggested by comparing median compensation figures at 6 years from starting practice in 2011^5^ to the median in 2011^6^ and consonant with 2004 data from MGMA [17]. Therefore, incomes are not necessarily related to seniority. In our sample, relatively more physicians are participants in the latter portion of their careers. Other factors that may underlie the inverse relationship of higher incomes and less mission participation are not ruled out by our analysis.

In order of importance, respondents indicate that time, work situation and family constraints exceed direct expenses as the primary reasons for not selecting STMMs among the options for volunteer activity. Reciprocally, the act of participation is unimpeded by these factors, implying that situational and emotional drivers outweigh innate demographic features in predicting who participates in STMMs.

It is perhaps somewhat counterintuitive to find in our sample that physician STMM participation was not predicted by religion despite the many faith-based organizations sponsoring STMMs readily found online. The 21% of respondents reporting no religious affiliation in this predominately Christian yet fundamentally tolerant country is in keeping with the reported trend towards non-affiliation with religions in the West and the opposite movement in the developing world [18]. Rather than religion as a prime driver of physician involvement in this activity, the decision to participate may be more closely related to tenets of medical oath than to tenets of faith, or to the degree of religiosity rather than the particular faith identification. The incomplete profile that emerges in our study falls short of shining a spotlight on the key impulse to participate. Our results do suggest that acting on the motivation is extensively regulated by the physical, professional, family and opportunity cost modifiers discussed.

A large majority of Hispanic physicians went to Latin American countries, but the same was true for black or African American physicians and non-Hispanic whites. Asians did lean towards Asia, so the attraction to ethnic roots or giving back to one’s heritage is mixed. It could be seen as encouraging that there is such limited attraction bias in that these donors of time and skill may be flexible enough to go where the need or best likelihood of impact calls them rather than the destination being determined solely by their ethnic ties or donor preferences [19]. A perhaps more likely alternative explanation for the Latin American mission target predominance would be the proximity to the US resulting in less travel cost and time, more of the time away being time on the ground working, and perhaps a longer cumulative experience with Latin American communities over time. Such a conclusion could be attractive were a similar regional connection to be found in future research for European and Australian physicians that do STMMs.

The limitations in our profile of the STMM physician begin with the relatively small sample size that is typical of email blast surveys. All such surveys are subject to response bias, and it is reasonable to anticipate that a survey on volunteerism risks more responses from those who do volunteer. In addition, one-sample chi square testing indicated incomplete external validity of our sample to the US physician population. The models are not powered to mitigate the effects of internal migration, change in marital status, practice situation or religion. Religion was not explored at the denominational level in our survey. Neither the effect of geographic variability in income nor the effect of dual income households on propensity to participate were assessed. Controlling for a high number of independent variables may induce insensitivity for all but the most robust relationships in small samples. A link between “who” to “why” may be unknowable and limited to inference. For all these reasons, applying our interpretations in the course of policy formulation requires prudence.

Narrative collections from STMM participants could be helpful to the bridge the gap between what demographic and economic data can tell us and the conscious process of a “go” decision, and thus from the “who” to the “why”. Narratives might also reveal how closely the personal reasons for attending in STMMs reflect the concepts related to the Public Goods, Private Consumption and Investment Exchange theories of philanthropy, and how the reward of “warm glow” may relate to opportunity costs that physicians are willing to trade for it [7, 10, 20].

## Conclusions

Respondents to the PGBS have documented that while there is no defined subset of the population that dominates the STMM participation, the participant is more likely to be a surgeon, anesthesiologist or pediatrician, married and in the latter portion of one’s career. The participant characterization may be extended by the influence of modes; the doctor has most likely reached an annual personal income level of 200–250 K USD with no children in the home. These attributes may render the STMM participant less cost sensitive and more likely to repeat STMM participation. Non-participation is more attributable to physical or social limitations or a sense that one’s specialty is not a fit for STMMs than direct costs, although higher incomes may dissuade participation due to larger opportunity cost. Latin American countries have been the targets of most US-sourced STMMs regardless of race or ethnicity except that most Asian physicians go to Asian destinations. Skilled and structured interrogatives with STMM physicians at various stages should add much value to understanding how the choice to participate is made.

## Additional file

Additional file 1: Physician’s Giving Back Survey. (PDF 265 kb)

## Abbreviations

DO: Medical degree, Doctor of Osteopathic Medicine
IMG: International medical graduate
LMIC: Lower and middle income countries
MD: Medical degree, Medicinae Doctor, Doctor of Medicine
MGMA: Medical group management association
PGBS: Physicians’ giving back survey
STMM: Short-term medical mission

## Footnotes

1 World Bank list of economies, February 2014, siteresources.worldbank.org/DATASTATISTICS/Resources/CLASS.XLS

2 “US MD” refers to physicians who received their Doctor of Medicine degree from a U.S. allopathic medical school. “DO” refers to physicians who received a Doctor of Osteopathy degree from a US-sanctioned osteopathic medical school. International medical graduate (IMG) refers to an individual with an MD or equivalent degree who graduated from a medical school outside the United States and its territories or Canada, including U.S. citizens who have attended such medical schools abroad. To be eligible for licensure and practice in the United States, all IMGs must have completed accredited graduate training in the United States.

3 American Medical Association (AMA) 2011 Physician Master File (data as of Dec. 31, 2010).

4 The Northeast includes nine states: Connecticut, Maine, Massachusetts, New Hampshire, Rhode Island, Vermont, New Jersey, New York and Pennsylvania; the Midwest region includes twelve states: Illinois, Indiana, Iowa, Kansas, Michigan, Minnesota, Missouri, Nebraska, North Dakota, Ohio, South Dakota and Wisconsin; the Southern region includes sixteen states: Florida, Georgia, Maryland, North Carolina, South Carolina, Virginia, West Virginia, Delaware, Alabama, Kentucky, Mississippi, Tennessee, Arkansas, Louisiana, Oklahoma and Texas; the Western region of the United States includes thirteen states: Montana, Wyoming, Colorado, New Mexico, Idaho, Utah, Arizona, Nevada, Washington, Oregon California, Alaska and Hawaii.

5 PayScale Human Capital. Posted on September 16, 2011 at www.physician-salary.org, Accessed 18 July 2015.

6 Physician Compensation and Production 2012 Report Cased on 2011 Data. Medical Group Management Association Datadrive Accessed 18 July 2015
