# Supplementary material for: Demographic profile of physician participants in short-term medical missions
Source: BMC Health Serv Res. 2016 Dec 7;16:682. doi: 10.1186/s12913-016-1929-x (PMC5142324; doi:10.1186/s12913-016-1929-x)
Supplement: Additional file 1: — Physician’s Giving Back Survey. (PDF 265 kb) [file 12913_2016_1929_MOESM1_ESM.pdf]

Physicians "Giving Back" Survey

Americans are generous people and that includes American doctors. Physicians often give their free time for teaching and supporting causes and organizations. Some US physicians provide free ("pro-bono") medical care to the needy domestically and/or go to developing countries to care for the poor. Such activities go beyond other cash donations to charities.

This instrument is designed to gather information on physician preference in choices of pro-bono activities, their prevalence and a few details on some activities. The research is part of a PhD dissertation with the University of Maastricht Graduate School of Governance/UNU-MERIT. As a physician, however you express your generosity, your anonymous response to the survey is critical to the analysis. Completion should take 2-15 minutes depending on your experiences.

For each 500 responses completed, $1000 will be donated to Doctors Without Borders, Habitat for Humanity or Grameen Foundation USA (in rotating order). Help drive the numbers!

You may also request to receive the data compilation and analysis at the completion of the project.

Paul H. Caldron, DO, FACP, FACR, MBA
4550 E. Bell Rd, Suite 172
Phoenix, Arizona 85032 USA
pcaldron@global.t-bird.edu

***1. I am a physician (MD/DO) who resides (resided) and practice (practiced) principally in the US and have completed my general and specialty training:** *(If you are still in clinical or supervised training, please answer "No".)*

◯ Yes

◯ No

***2. My professional volunteerism and giving activity since finishing all my training has been expressed in the following ways (please check all that apply):**

- [ ] I am not yet in a position to participate in any volunteer or giving activities
- [ ] Monetary or donations-in-kind to causes and/or organizations
- [ ] Pro-bono medical care in local / domestic clinics or outreaches (sports physicals, kids' camps, etc.)
- [ ] Time devoted to the organized activities of patient support organizations (Arthritis Foundation, Cancer Society, etc.)
- [ ] No activities so far since I have been in medical practice
- [ ] There is a system in my practice to accommodate those who have limited means to pay
- [ ] Pro-bono medical care in developing countries (short-term medical missions)
- [ ] Pro-bono teaching to medical personnel
- [ ] I choose not to participate in any such activities
- [ ] Other (please specify)

3. My pro-bono (unpaid) teaching activities since I have been in practice have included the following settings (please check all that apply):

- [ ] None
- [ ] Medical students, residents, fellows, nurses or ancillary service persons in an office setting
- [ ] Medical students, residents, fellows, nurses or ancillary service persons in a hospital setting
- [ ] Health – related teaching to civic organizations or general public
- [ ] Health – related teaching to public or private schools

4. In my individual practice, the estimated annual amount of money that is intentionally left uncollected from persons with limited means to pay (highest year estimate) is:

- ( ) None
- ( ) $1-999
- ( ) $1,000 – $4,999
- ( ) $5,000 – $19,999
- ( ) $20,000 – $50,000
- ( ) Over $50,000
- ( ) No way to know because I work purely on salary
- ( ) No way to know because I work in a public institution
- ( ) Other (please specify)

5. My pro-bono local / domestic medical services have included (please check all that apply):

- [ ] None
- [ ] Local free / sliding scale clinic after my usual practice hours
- [ ] Non- paid screening physicals, team or group doctor for schools or other organizations
- [ ] Local / domestic disaster response wherein I provide free medical services
- [ ] Domestic short-term mission work for which I must schedule time away from my practice
- [ ] Other domestic pro-bono medical services

***6. My pro-bono participation in a short-term medical mission in a developing country has been:**

- ○ None
- ○ One time
- ○ More than once

*7. I have not participated in a short-term mission abroad because of (check all that apply):

- [ ] Belief that the activity is not cost effective
- [ ] Belief that the activity is potentially harmful
- [ ] My specialty is not suitable for remote, low-technology environments
- [ ] Cost
- [ ] My own physical or social limitations
- [ ] No interest
- [ ] Never considered
- [ ] I previously participated in full-time medical mission work abroad
- [ ] Belief that the activity is ineffective
- [ ] Other

Where in the US were you living at the time of the short-term medical mission?

***8. State:**

9. City/town population:

***10. What was your practice situation at the time of the mission?**

***11. How many years had you been in practice at the time of the mission?**

***12. In what country was your mission?**

***13. In what year was your mission?**

***14. How long was your mission (number of days away)?**

***15. Your age at time of mission:**

***16. What was your civil status at the time of the mission?**

17. If you had children (biologic and/or adopted), how many were living at home at the time of the mission?

***18. What was the focus of the mission's medical service?**

- ◯ Medical only
- ◯ General Surgical
- ◯ Specialty Surgical
- ◯ Mixed Medical and Surgical
- ◯ Other (please specify)

Mission Expenses: Most short-term medical mission organizers require that physician participants pay a fee that covers mission costs including lodging, meals, ground transportation and support for mission staff, supplies and other fees. Individuals must usually arrange and cover their own air travel to the receiving country. Further, some physicians and surgeons donate supplies, equipment, implantable devices or hardware, etc. at their own personal expense.

To the best of your immediate recollection, for your mission, your expenses were:

***19. Airfare:**

[ ]

***20. Mission organization fees paid:**

[ ]

***21. Additional personal expenses for donated supplies, equipment, implantable devices or hardware, etc.: (Provide your best immediate estimate.)**

[ ]

Now, please consider the time you were away from your practice for the mission and your stated income range at the time of the mission. Estimate the total earnings that you could have earned if you had not gone on the mission (taxable income as well as retirement funding or other deferments):

***22. What were the total earnings you could have earned? (Provide your best immediate estimate.)**

[ ]

***23. What percentage of your medical mission total expenses was deductible from your Federal taxable income?**

- ○ 100%
- ○ Other % (please specify)

[ ]

***24. In the year you went on your short-term medical mission to a developing country, what was your total annual income as reported on your Federal Tax return in the <u>calendar year prior</u> to the mission? (Provide your best immediate estimate.)**

[ ]

25. Would you consider going on additional short-term medical missions?

- ○ Yes
- ○ No

26. If you answered “No”, why not? (please check all that apply)

- [ ] Cost is prohibitive
- [ ] I sacrificed too much income going on the mission
- [ ] My own physical or social limitations
- [ ] My specialty is not suitable for remote, low-technology environments
- [ ] Too much time away from spouse and/or family
- [ ] Belief that the activity is not cost effective
- [ ] Belief that the activity is potentially harmful
- [ ] Belief that the activity is ineffective
- [ ] Other

***27. Number of short-term medical missions**

***28. Please enter information about your missions:**

	Year of mission	Country of mission	Number of days on mission
Mission 1			
Mission 2			
Mission 3			
Mission 4			
Mission 5			
Mission 6			
Mission 7			
Mission 8			
Mission 9			
Mission 10			

29. If you've been on more than 10 medical missions, please enter additional information in the box.

Where in the US were you living at the time of your FIRST short-term medical mission?

***30. State:**

[ ]

31. City/town population:

[ ]

***32. What was your practice situation at the time of the FIRST mission?**

[ ]

***33. How many years had you been in practice at the time of the FIRST mission?**

[ ]

***34. Your age at time of FIRST mission:**

[ ]

***35. What was your civil status at the time of the FIRST mission?**

[ ]

36. If you had children (biologic and/or adopted), how many were living at home at the time of the FIRST mission?

[ ]

***37. What was the focus of the FIRST mission's medical service?**

- ◯ Medical only
- ◯ General Surgical
- ◯ Specialty Surgical
- ◯ Mixed Medical and Surgical
- ◯ Other (please specify)

[ ]

Mission Expenses: Most short-term medical mission organizers require that physician participants pay a fee that covers mission costs including lodging, meals, ground transportation and support for mission staff, supplies and other fees. Individuals must usually arrange and cover their own air travel to the receiving country. Further, some physicians and surgeons donate supplies, equipment, implantable devices or hardware, etc. at their own personal expense.

To the best of your immediate recollection, for your FIRST mission, your expenses were:

***38. FIRST medical mission airfare:**

[ ]

***39. FIRST medical mission organization fees paid:**

[ ]

***40. FIRST medical mission additional personal expenses for donated supplies, equipment, implantable devices or hardware, etc.: (Provide your best immediate estimate.)**

[ ]

Now, please consider the time you were away from your practice for your FIRST mission and your stated income range at the time of the mission. Estimate the total earnings that you could have earned if you had not gone on the mission (taxable income as well as retirement funding or other deferments):

***41. What were the total earnings you could have earned? (Provide your best immediate estimate.)**

[ ]

***42. What percentage of your FIRST medical mission total expenses was deductible from your Federal taxable income?**

- ○ 100%
- ○ Other % (please specify)

[ ]

***43. In the year you went on your FIRST short-term medical mission to a developing country, what was your total annual income as reported on your Federal Tax return in the calendar year prior to the FIRST mission? (Provide your best immediate estimate.)**

[ ]

Where in the US were you living at the time of your MOST RECENT short-term medical mission?

***44. State:**

45. City/town population:

***46. What was your practice situation at the time of the MOST RECENT mission?**

***47. How many years had you been in practice at the time of the MOST RECENT mission?**

***48. Your age at time of MOST RECENT mission:**

***49. What was your civil status at the time of the MOST RECENT mission?**

50. If you had children (biologic and/or adopted), how many were living at home at the time of the MOST RECENT mission?

***51. What was the focus of the MOST RECENT mission's medical service?**

- ○ Medical only
- ○ General Surgical
- ○ Specialty Surgical
- ○ Mixed Medical and Surgical
- ○ Other (please specify)

Mission Expenses: Most short-term medical mission organizers require that physician participants pay a fee that covers mission costs including lodging, meals, ground transportation and support for mission staff, supplies and other fees. Individuals must usually arrange and cover their own air travel to the receiving country. Further, some physicians and surgeons donate supplies, equipment, implantable devices or hardware, etc. at their own personal expense.

To the best of your immediate recollection, for your MOST RECENT mission, the expenses were:

***52. MOST RECENT medical mission airfare:**

***53. MOST RECENT medical mission organization fees paid:**

***54. MOST RECENT medical mission additional personal expenses for donated supplies, equipment, implantable devices or hardware, etc.: (Provide your best immediate estimate.)**

Now, please consider the time you were away from your practice for your MOST RECENT mission and your stated income range at the time of the mission. Estimate the total earnings that you could have earned if you had not gone on the mission (taxable income as well as retirement funding or other deferments):

***55. What were the total earnings you could have earned? (Provide your best immediate estimate.)**

***56. What percentage of your MOST RECENT medical mission total expenses was deductible from your Federal taxable income?**

- 100%
- Other % (please specify)

***57. In the year you went on your MOST RECENT short-term medical mission to a developing country, what was your total annual income as reported on your Federal Tax return in the calendar year prior to the MOST RECENT mission? (Provide your best immediate estimate.)**

***58. In what year were you born?**

***59. What is your gender?**

- Female
- Male

60. Are you of Hispanic, Latino or Spanish origin?

- Yes
- No

61. What is your race? For purposes of this question, persons of Spanish/Hispanic/Latino origin may be any race.

- White
- Black or African American
- American Indian or Alaska Native
- Asian
- Native Hawaiian or other Pacific Islander
- Other (please specify)

***62. How many years have you been in practice?**

***63. What is your principal practice specialty?**

***64. Were you born in the USA?**

- Yes
- No

65. If not in the USA, in what country were you born?

***66. Did you attend medical school in the USA?**

- ◯ Yes
- ◯ No

67. If not in the USA, in what country did you attend medical school?

[ ]

***68. Did you do most of your postgraduate training (internship, residency, fellowship) in the USA?**

- ◯ Yes
- ◯ No

69. If not in USA, in what country did you do most of your post graduate training?

[ ]

***70. What is your medical degree?**

[ ]

71. What is your current civil status?

***72. The total number of children (biologic and/or adopted) you have now is:**

73. What is your religious affiliation?

Where in the US do you live now?

***74. State:**

75. City/town population:

***76. What is your current practice situation?**

77. What is the highest annual income you ever reported on your Federal tax return? (Provide your best immediate estimate.)

Thank you so much for taking the time to complete this survey and assisting in my research on physician volunteerism. Your input is invaluable in the further understanding and description of this activity.

To request the data compilation and analysis at the completion of the project, please contact me at pcaldron@global.t-bird.edu. You may also contact me at this email if you have questions or wish to share related information.

Thank you,

Dr. Paul H. Caldron
4550 E. Bell Rd, Suite 172
Phoenix, Arizona 85032 USA
pcaldron@global.t-bird.edu
